# Core Needle Biopsy Accuracy for Androgen Receptor Expression in Invasive Breast Cancer

**DOI:** 10.1055/s-0043-1772486

**Published:** 2023-10-16

**Authors:** Marcelle Morais dos Santos, Antonio Luiz Frasson, Vinicius Duval da Silva, Aluísio de Castro Antunes Maciel, Guilherme Watte, Gustavo Werutsky, Tomás Reinert, André Poisl Fay

**Affiliations:** 1 Department of Breast Surgery, Hospital São Lucas, Pontifícia Universidade Católica do Rio Grande do Sul, Porto Alegre, RS, Brazil; 2 Department of Pathology, Hospital do Câncer de Barretos, Barretos, SP, Brazil; 3 Department of Pathology, Hospital São Lucas, Pontifícia Universidade Católica do Rio Grande do Sul, Porto Alegre, RS, Brazil; 4 Department of Medical Oncology, Hospital São Lucas, Pontifícia Universidade Católica do Rio Grande do Sul, Porto Alegre, RS, Brazil; 5 School of Medicine, Pontifícia Universidade Católica do Rio Grande do Sul, Porto Alegre, RS, Brazil; 6 Oncoclínicas, Porto Alegre, RS, Brazil

**Keywords:** breast cancer, androgen receptor, core needle biopsy, immunohistochemistry, biomarkers, câncer de mama, receptores androgênicos, biópsia com agulha de grande calibre, imuno-histoquímica, biomarcadores

## Abstract

**Objective** Breast cancer (BC) biomarkers, such as hormone receptors expression, are crucial to guide therapy in BC patients. Antiandrogens have been studied in BC; however, limited data are available on androgen receptor (AR) expression test methodology. We aim to report the core needle biopsy (CNB) accuracy for AR expression in BC.

**Methods** Patients diagnosed with stage I-III invasive BC from a single institution were included. Androgen receptor expression was evaluated by immunohistochemistry (IHC) using 1 and 10% cutoff and the AR expression in surgical specimens (SS) was the gold standard. Kappa coefficients were used to evaluate the intraprocedural agreement.

**Results** A total of 72 patients were included, with a mean age of 61 years old and 84% were Luminal A or B tumors. The prevalence of AR expression in all BC samples was 87.5% using a cutoff ≥ 10% in SS. With a cutoff value ≥ 1%, CNB had an accuracy of 95.8% (Kappa value = 0.645; 95% confidence interval [CI]: 0.272–1.000; *p* < 0.001) and 86.1% (Kappa value = 0.365; 95% CI: 0.052–0.679; *p* < 0.001) when ≥ 10% cutoff was used for AR positivity. Androgen receptor expression in CNB (cutoff ≥ 1%) had a sensitivity of 98.5%, specificity of 60%, positive predictive value of 97.0%, and a negative predictive value of 76.9% in the detection of AR expression in SS.

**Conclusion** Core needle biopsy has good accuracy in evaluating AR expression in BC. The accuracy of CNB decreases with higher cutoff values for AR positivity.

## Resumo

**Objetivo** Biomarcadores, como a expressão de receptores hormonais, são cruciais para guiar a terapia de pacientes com câncer de mama. Apesar de ter sido estudado, poucos dados estão disponíveis sobre o método de testagem. Buscamos avaliar a precisão da biópsia com agulha de grande calibre (CNB, na sigla em inglês) para a expressão de receptores androgênicos (AR, na sigla em inglês) no câncer de mama.

**Métodos** Foram incluídos pacientes de uma única instituição diagnosticados com câncer de mama invasivo estágio I-III. A expressão de AR foi avaliada por imunohistoquímica, com valores de cutoff de 1 e 10%. A expressão de AR em espécimes cirúrgicos foi o padrão ouro. O coeficiente Kappa foi usado para avaliar a concordância entre procedimentos.

**Resultados** Foi incluído um total de 72 pacientes, com idade média de 61 anos; 84% eram tumores luminais A ou B. A prevalência da expressão de AR em todas as amostras foi de 87.5%, com cutoff ≥ 10%. Com um valor de cutoff ≥ 1%, a CNB teve precisão de 95.8% (Kappa = 0.64; intervalo de confiança [IC] 95%: 0.272–1.000; *p* < 0.001) e 86.1% (Kappa = 0.365; CI95%: 0.052–0.679]; *p* < 0.001) quando um cutoff ≥ 10% foi usado para AR positivo. A expressão de AR na CNB (cutoff ≥ 1%) teve a sensibilidade de 98.5%, especificidade de 60%, valor preditivo positivo de 97.0% e valor preditivo negativo de 76.9% na detecção.

**Conclusão** |Biópsia com agulha de grande calibre tem uma boa precisão em avaliar a expressão de AR no câncer de mama. A precisão do método cai com valores elevados de cutoff para AR positivo.

## Introduction

Breast cancer (BC) is a heterogeneous disease. Immunohistochemistry (IHC) is the routine pathological technique used to evaluate hormone receptor (HR) status, HER2 expression, among other markers to better stratify BC subtypes. 1 2 Immunohistochemistry performed in the diagnostic preoperative core needle biopsy (CNB) samples is critical to define whether neoadjuvant therapy is necessary and the type of drug regimen to be used. 3 4 In case of pathologic complete response, which is common in more aggressive BC subtypes after neoadjuvant treatment, the CNB specimen can be the only biological material left for further biomarkers analysis. However, IHC assessment in CNB samples may be less reliable than in surgical specimens (SS) due to a variety of factors, including the relatively smaller sample size and tumor heterogeneity. 5

Androgen receptor (AR) expression in breast cancer is often associated with better prognostic tumors, was identified as a subtype of triple negative breast cancer (TNBC) and as a potential therapeutic target, especially in TNBC. 6 7 8 9 10 11 12 13 14 Androgen receptor-targeted agents such as bicalutamide, enzalutamide and abiraterone acetate have shown promising preliminary results in advanced BC 15 16 17 18 and there are currently ongoing trials evaluating the role of antiandrogens in HR-positive and TNBC, 19 20 21 although CNB accuracy for AR expression in invasive BC has not been evaluated in previous studies.

The primary goal of the present study is to describe the CNB accuracy for the evaluation of AR expression in BC in a Brazilian population.

## Methods

We conducted a cross-sectional study to evaluate biomarkers expression in BC specimens. Clinical data from consecutive patients diagnosed with invasive BC treated in the Surgical Breast Unit of Hospital São Lucas of the Pontifícia Universidade Católica do Rio Grande do Sul (PUCRS) in Brazil, from March 2017 to March 2018, were retrospectively collected. All patients underwent CNB and subsequently had surgical procedure in our institution. Women ≥18 years old who have agreed to participate in our study and have signed a written informed consent were included in the analysis. Patients who received neoadjuvant treatment and for whom material from surgical specimens was sufficient for AR, estrogen receptor (ER) and progesterone receptor (PR) status evaluation were also included. Neoadjuvant treatment may impair the biomarker analysis once intratumoral heterogeneity and different responses of cellular clones may result in discordant IHC. The exclusion criteria were in situ or microinvasive BC, because this subgroup of carcinomas was not included in the trials that evaluated the role of anti-androgens as a potential therapeutic target. We also excluded multicentric or multifocal tumors to make sure that we analyzed the same tumor by core biopsy and surgical specimen, to evaluate the accuracy properly. The Local Ethics Committee approved the study under the Certificate of Ethical Assessment (CAAE) registration number 60989316.0.0000.5336. Clinical data such as patient age, menopausal status, surgical treatment type, neoadjuvant treatment history, number of fragments obtained from CNB and main pathological findings from both CNB and SS, which included tumor size, tumor grade, and tumor biomarkers expression, were collected through retrospective review of medical records and pathologic reports.

Percutaneous CNB was performed under local anesthesia with a semiautomated biopsy gun with a 14 Gauge (14-G), 10 cm long needle. A mean of 6 core samples per lesion (range 2–11) were obtained. In 58 cases, ultrasound guidance was performed and in 14 cases the guidance was performed by the assistant physician, because the tumor was easily palpable. Fragments of CNB were placed immediately into an adequate volume of 10% buffered formaldehyde. A minimum fixation time of 6 hours and a maximum of 72 hours were ensured, according to the American Society of Clinical Oncology and the College of American Pathologists (ASCO/CAP) guidelines 22 prior to tissue processing and paraffin embedding.

The tumor was incised after painting of the surgical margins to facilitate fixation of breast conserving surgery specimens and the mastectomy specimens were inked and cut into 1-cm-thickness slices before fixation. Sampled tissue blocks were fixed in an adequate volume of 10% buffered formaldehyde for between 12 and 24 hours, processed in a Shandon Excelsior ES (ThermoFischer Scientific, Waltham, MA, USA) and then embedded in paraffin. Three-μm-thickness paraffin sections were cut for hematoxylin-eosin staining and IHC analysis.

The evaluation of AR, PR, and ER by IHC was performed using the following validated primary antibodies: anti-AR, clone AR441, dilution 1:100 (Biocare, Monoclonal Mouse Anti-Human Androgen Receptor); anti-ER, clone EP1, ready-to-use (Dako, Monoclonal Rabbit Anti-Human Estrogen Receptor α) and anti-PR, clone 636, ready-to-use (Dako, Monoclonal Mouse Anti-Human Progesterone Receptor). Material was processed in an automated system for immunohistochemical reactions (EnVision Flex/ HRP, Agilent, USA) and IHC analysis was done by a trained pathologist with qualitative and semiquantitative image analysis. The IHC uses labeled antibodies to localize specific activations of antigen proteins in the tissue sections. The qualitative image analysis consists in simple observation of the presence and darkness of specific stains within the tissue, while the semiquantitative method estimates the quantity of proteins on chromogen-labeled immunohistochemical (IHC) tissue sections via computer-aided methods. Material from both CNB and SS were processed and analyzed in the same institution.

The ASCO/CAP guideline 22 was used to define dichotomy – define the cutoff for positivity and negativity, which recommends that ER and PR assays be considered positive if there are at least 1% positive tumor nuclei in the testing sample, in the presence of expected reactivity of internal (normal epithelial elements) and external controls. For AR expression, we used two cutoff values to consider positivity. First: ≥ 1% and second: ≥ 10%, due to different cutoffs used in previous studies. 23 24

Breast surgery was categorized as breast conserving surgery or mastectomy. The later includes simple mastectomy (without immediate reconstruction procedure), nipple sparing mastectomy, and skin-sparing mastectomy (both with immediate reconstruction procedure).

Surgical specimens were classified into molecular subtypes according to the 12th St. Gallen International Breast Cancer Conference [4] as: luminal A, luminal B (HER2 negative or HER2 positive), HER2 positive nonluminal (also known as HER2-enriched), and triple negative.

The primary endpoint was to describe the CNB accuracy for the evaluation of AR expression in BC by IHC. The secondary endpoint was to perform the same evaluation for ER and PR expression. Definitive histological diagnosis and biomarkers on SS served as the gold standard in subsequent analyzes.

Sample size calculation was performed with R/R Studio software. It was estimated that, in different Kappa coefficients, 80 patients would provide a margin of error between 0.10 and 0.11 for agreement evaluation. We also took into consideration the consecutive sample available with AR analysis in our institution, a limited sample. Data were presented as mean ± standard deviation (SD) or frequency and percentage. Kappa coefficients were used to evaluate the intraprocedural agreement. Its interpretation was conducted based on the following parameters: Kappa of 0.01 indicates “poor” agreement; Kappa ranges from 0.01 to 0.20 indicate “slight” agreement; Kappa from 0.21 to 0.40 indicate “fair” agreement; Kappa from 0.41 to 0.60 indicate “moderate” agreement; Kappa from 0.61 to 0.80 indicate “substantial” agreement, and Kappa ranging from 0.81 to 1.00 indicate “almost perfect” agreement. 25 Using SS as the gold standard, sensitivity, specificity, positive predictive value (PPV) and negative predictive value (NPV) for CNB were calculated for each of the IHC analysis: AR, ER, and PR expression. In all cases, *p-* values < 0.05 were considered statistically significant. The statistical analysis was performed using IBM SPSS Statistics for Windows version18 (IBM Corp., Armonk, NY, USA).

## Results

The study included 72 patients with a mean age of 61 years old; 78% were postmenopausal. The mean number of samples in each CNB was 6 ± 2; 51 (71%) patients underwent breast conserving surgery and 21 (29%) underwent mastectomy. The majority had Luminal A (44%) and Luminal B HER2 negative (40%) tumors. There were no HER-enriched breast carcinomas in our sample. For all patients included independent of BC subtype, the frequency of AR expression was 87.5% using a cutoff point of ≥ 10% and 94.4% when the cutoff point was ≥ 1%. Patient characteristics are summarized on Table 1 .

**Table 1:** Patient characteristics

Parameter	*n* = 72
Age (years old)	61 ± 12
Menopausal status	
Pre- or peri-	16 (22)
Post-	56 (78)
Mean number of core biopsies	6 ± 2
Surgical treatment	
Axillary surgery	
No	1 (1)
Axillary dissection	15 (21)
Sentinel lymph node biopsy	56 (78)
Breast surgery	
Breast conserving surgery	51 (71)
Mastectomy	21 (29)
Histologic type	
Invasive carcinoma NST	62 (86)
Invasive lobular carcinoma	6 (8)
Mucinous carcinoma	3 (4)
Papillary carcinoma	1 (1)
Tumor size in mm	16 [10–24]
Tumor grade	
I	21 (30)
II	35 (49)
III	15 (21)
Molecular subtype	
Luminal A	32 (44)
Luminal B HER2 negative	29 (40)
Triple negative	07 (10)
Luminal B HER2 positive	4 (6)
Pathologic N stage	
N0	46 (69)
N1	13 (19)
N2	2 (3)
N3	6 (9)
Neoadjuvant chemotherapy	17 (23)
Neoadjuvant hormonal therapy	2 (3)

Abbreviations: HER2, human epithelial growth factor receptor 2; NST, no special type.

Data were presented as mean ± standard deviation, median (IQR – interquartile range) or No. (%).

The AR expression accuracy was 95.8% using a cutoff ≥ 1% (Kappa = 0.645; 95% confidence interval [CI]: 0.272–1.000; *p* < 0.001). A lower accuracy of 86.1% for AR expression was found when using a cutoff ≥ 10% (Kappa = 0.365; 95%CI: 0.052–0.679; *p* < 0.001). For hormone receptor status, the accuracy of ER expression was 943% (Kappa = 0.636; 95%CI: 0.309–0.964; *p* < 0.001), and 88.5% for PR expression (Kappa = 0.643; 95%CI: 0.417–0.869; *p* < 0.001), which were similar to AR. Table 2 shows results for the diagnostic capability of CNB to evaluate AR, ER, and PR expression. With a cutoff value ≥ 1%, the AR expression in CNB samples had a sensitivity of 98.5%, specificity of 60%, PPV of 97.0%, and NPV of 76.9% in the detection of AR expression in SS. With a cutoff value ≥ 10%, the AR expression in CNB samples had a sensitivity of 92%, specificity of 44.4%, PPV of 92% and NPV of 44.4% in the detection of AR expression in SS.

**Table 2:** Diagnostic capability of core biopsy using surgical specimens as the gold standard

Core biopsy	Surgical specimen		Sensitivity	Specificity	PPV	NPV	Accuracy
	Positive	Negative					
Estrogen receptor			98.41%	57.14%	95.38%	80.00%	94.29%
≥ 1%	62	02					
< 1%	01	04					
Progesterone receptor			94.55%	66.67%	91.23%	76.92%	88.57%
≥ 1%	52	05					
< 1%	03	10					
Androgen receptor			98.51%	60.00%	97.06%	76.92%	95.83%
≥ 1%	66	02					
< 1%	01	03					
Androgen receptor			92.06%	44.44%	92.06%	44.44%	86.11%
≥ 10%	58	05					
< 10%	05	04					

Abbreviations: NPV, negative predictive value; PPV, positive predictive value.

The prevalence of ER expression was 91.3% with the cutoff point ≥ 1%. The ER in CNB samples had a sensitivity of 98.4%, specificity of 57.1%, PPV of 95.3% and NPV of 80% in the detection of ER expression in SS. The prevalence of PR expression was 78,5% with the cutoff point ≥ 1%. The PR expression in CNB samples had a sensitivity of 94.5%, specificity of 66.6%, PPV of 91.2%, and NPV of 76.9% in the detection of PR expression in SS. Table 3 shows the distribution of AR positivity in CNB and in SS by BC subtypes.

**Table 3:** Distribution of AR positivity in core biopsy and surgical specimens by molecular subtypes

Molecular subtype	AR core biopsy		AR surgical specimens	
	≥1%	≥10%	≥1%	≥10%
Luminal A ( *n* = 32)	32	31	32	31
Luminal B HER2 negative ( *n* = 29)	28	25	28	25
Triple negative ( *n* = 7)	03	03	04	03
Luminal B HER2 positive ( *n* = 4)	04	04	04	04

Abbreviation: HER2, human epithelial growth factor receptor-type 2.

## Discussion

In our analysis, we demonstrate that CNB accuracy for AR expression is high, but it decreases when a higher cutoff value (≥ 10%) is used. Five (6.9%) of the 72 patients had AR negative in CNB and positive in SS (2 received neoadjuvant chemotherapy). On the other hand, 5 (6.9%) patients who had positive AR in CNB turned out to have a negative result in the SS (1 received neoadjuvant chemotherapy). Therefore, it is expected that a small number of patients will have discordant results possibly due to intratumoral heterogeneity and exposure to neoadjuvant chemotherapy. 26

Estrogen receptor and PR expression remain the most important biomarkers in breast cancer over the last decades, even though the definition of the optimal threshold to define HR positivity remain controversial. Recently, the ASCO/CAP guideline for estrogen and progesterone receptor testing in breast cancer was updated [1]. Results with 1 to 10% of cells staining positive should be reported using a new category (“low positive”). The limited data on endocrine therapy benefit in this subgroup tailored the new reporting recommendation, but it should not change the patient eligibility for endocrine therapies. The same issue is important regarding the evaluation of the AR expression.

The AR is a steroid-hormone activated transcription factor belonging to the nuclear receptor superfamily, which also includes the ER and PR. The AR pathway is associated with regulation of normal breast development, as it appears to balance the estrogen-induced cell proliferation, and also with breast tumor carcinogenesis. 27 28 29 30 The precise mechanism and clinical implications of AR action in BC remains poorly understood. Several studies support the prognostic role of AR, with different mechanisms dependent on coexpression of HR or HER2 amplification. 31 32 33 A subset of TNBC (the luminal androgen receptor [LAR]) is dependent upon androgen signaling for growth and therapies that inhibit androgen signaling have been tested with promising results. 34 35 36

Neoadjuvant therapies have been increasingly used in breast oncology not only as a clinical tool to allow tumor downstaging and less extensive surgeries, but also as a scientific tool to the evaluation of biomarkers and development of targeted therapies. In this case, the systemic therapy that will be offered will depend on disease staging and tumor biomarkers, such as HR evaluated on CNB.

Androgen receptor expression was associated with chemoresistance in TNBC and endocrine therapy resistance in Luminal tumors. 37 38 39 40 41 Mohammed et al. have shown that TNBC AR+ had a lower rate of pathological complete response (pCR, defined as no invasive residual disease in the breast or nodes after neoadjuvant chemotherapy) compared with TNBC AR- (24.1 versus 60%, respectively; *p* < 0.01). 42

In our study, the prevalence of AR expression was 87.5% in SS using a cutoff ≥ 10% and 94.4% with a cut-off ≥1%, which are similar to previous data. 28 43 The distribution of AR expression according to BC subtypes using a cutoff ≥ 10% were: 96.8% in Luminal A, 86.2% in Luminal B HER2 negative, 42.8% in TNBC, and 100% in Luminal B HER2 positive. A previous study identified AR expression in a range of 50 to 90% in Luminal A and B, 50 to 60% in HER2-positive, and between 20 and 40% in TNBC. 43

Nonetheless, the AR expression in BC has no standard procedure and evaluation and results are variable depending on the cutoff levels for positivity (≥ 1%, ≥ 5% or ≥ 10% in IHC), 23 the antibody used in staining, and the methodology (if it was automated qualitative, semiquantitative, or quantitative image analysis. The papers available in the literature are very heterogeneous in this analysis). A recent study presented at ASCO 2020 corroborated the inconsistency in AR evaluation. 20 For example, a cutoff point ≥ 30% for AR IHC had the best concordance with LAR subtype (r = 0.6; *p* < 0.001).

Two ongoing studies [20,21] evaluating the role of antiandrogen drugs in a neoadjuvant setting selected patients to receive therapy with enzalutamide using AR positivity based on CNB IHC, each study using a different cutoff for AR positivity (1 and 10%), which clearly highlight the lack of consensus in methodology and the potential implications of trial results in practice.

Several studies have shown a high concordance between ER and PR status evaluated on CNB and SS. The concordance found in these studies ranged from 92 to 96% for ER and from 88 to 94% for PR. 44 45 46 47 Our analysis of CNB accuracy for ER and PR expression was similar to those of previous reports, 94.2% for ER and 88.5% for PR, which reinforces the quality of IHC methodology applied in our study.

The AR expression is usually not evaluated in current clinical practice but the increasing interest in this biomarker as a predictive and therapeutic target corroborates the need of an accurate evaluation and consensus regarding the threshold to define positivity as it may be used for BC classification subtype and therapeutic decision. It is also important to address the potential harm of indicating the use of antiandrogen drugs in false-positive cases. It includes adverse effects (AE) like headache, muscular weakness, and anxiety, mostly grade 1 or 2 (slight to mild), which are usually tolerable for the patients.

The present study has some limitations. We evaluated a relatively small sample size, which impair a sub-analysis in different BC subtypes, and there was a higher proportion of Luminal A and B tumors, which may impact the results of AR expression prevalence. Furthermore, we have included a subgroup of patients who have received neoadjuvant treatment (chemotherapy or endocrine therapy), which may impact biomarker analysis. Nevertheless, our study contributes to the very limited data regarding AR expression accuracy in terms of CNB and SS, also to add validity to our methodology, we performed analysis of ER and PR expression, which showed consistent accuracy results.

## Conclusion

Our study shows that evaluation of AR expression by IHC using CNB samples is feasible and has a high accuracy. Using a cutoff ≥ 10% for AR expression decreases the agreement between CNB and SS. This finding has implications for the pathological analysis of AR especially in clinical trials evaluating antiandrogen agents. 48
